# Surgical Management for a Case of Baastrup's Disease With Concomitant Multiple Lumbar Spondylolysis and Epidural Cyst

**DOI:** 10.7759/cureus.82764

**Published:** 2025-04-22

**Authors:** Kazunori Hayashi, Toru Tanaka, Akira Sakawa, Tsuneyuki Ebara, Hidetomi Terai

**Affiliations:** 1 Orthopedic Surgery, Osaka City Juso Hospital, Osaka, JPN; 2 Orthopedic Surgery, Osaka Metropolitan University Graduate School of Medicine, Osaka, JPN

**Keywords:** baastrup’s disease, direct repair of pars, lumbar interbody fusion, lumbar spinal stenosis, lumbar spondylolysis

## Abstract

Baastrup's disease is a degenerative condition characterized by the close approximation of adjacent spinous processes. Interspinous bursal cysts may extend into the epidural space, resulting in symptomatic central canal stenosis. Lumbar multiple spondylolysis, characterized by defects in more than one lumbar lamina, is a rare condition often associated with trauma or repetitive stress. This case report presents a 56-year-old male plumber who presented with low back pain and bilateral leg numbness. He had intermittent claudication at 500 m. Imaging revealed L4 spondylolytic spondylolisthesis, L5 spondylolysis, and Baastrup's disease at L3-L4 with an extradural cyst. He underwent L4-L5 posterior lumbar interbody fusion with cyst resection and direct repair of the L5 pars defect using a lamina hook system. Histopathology confirmed a synovial cyst. Symptoms resolved postoperatively, and he regained full mobility. At two years, imaging showed solid L4-L5 fusion with preserved L5-S1 motion. This case highlights the importance of limited fusion to avoid excessive segmental immobilization, with a combined fixation approach providing symptom relief and spinal stability.

## Introduction

Baastrup's disease, also referred to as "kissing spine syndrome," is a degenerative condition characterized by the close approximation of adjacent spinous processes. It typically affects the lumbar spine, with the L4-L5 level being the most common site of involvement [1,2]. This condition is frequently observed in individuals over the age of 70 years and is characterized by low back pain that is exacerbated by extension and alleviated by flexion [1]. In some cases, this can result in the formation of interspinous bursal cysts, which have been documented in 8.2% of patients presenting with back or leg pain [3]. In rare instances, less than 1%, these cysts may extend into the posterior epidural space, resulting in symptomatic central canal stenosis and necessitating intervention [4,5].

Lumbar multiple spondylolysis is a rare condition characterized by pars defects in more than one lumbar vertebra. The condition is more prevalent in males and is frequently linked to traumatic incidents or strenuous physical exertion [6,7]. Most cases involve two spinal levels, typically at L3-L4 and L4-L5 [6]. Sakai et al. reported five cases of multiple lumbar spondylolysis as observed through the analysis of 2000 CT scans, including asymptomatic subjects [8].

This case report presents a rare occurrence of concomitant lumbar spondylolysis and epidural cyst formation in a patient with Baastrup's disease, resulting in cauda equina and nerve root symptoms. The patient underwent posterior lumbar interbody fusion (PLIF) at the affected level and direct repair of the pars at the adjacent spondylolysis level.

This case study emphasizes the necessity of avoiding multiple interbody fusion, which has a relatively high complication rate, and preserving the mobile lumbar segment as much as possible. Fusion at the lumbar level resulting in Baastrup's disease leads to an early resolution of symptoms, whereas repair or bracing at the other level to prevent worsening of slippage or the development of interspinous cysts may achieve favorable clinical outcomes.

This article was previously presented as a poster at the Japanese Spinal Instrumentation Society or JSIS annual meeting on September 20, 2024.

## Case presentation

A 56-year-old male plumber presented with low back pain and bilateral numbness in the lower extremities. Symptoms began one year ago with spontaneous pain in the right anterior lower leg with gait, which spread to the left leg one month ago. He reported intermittent claudication at 500 m. Neurological examination was largely normal without muscle weakness, but the right femoral nerve stretch test was positive. Visual analog scale (VAS) scores were 1.5 for back pain, 3.8 for leg pain, and 3.2 for numbness; the Japanese Orthopedic Association (JOA) score for low back pain was 15/29.

**Figure 1:**
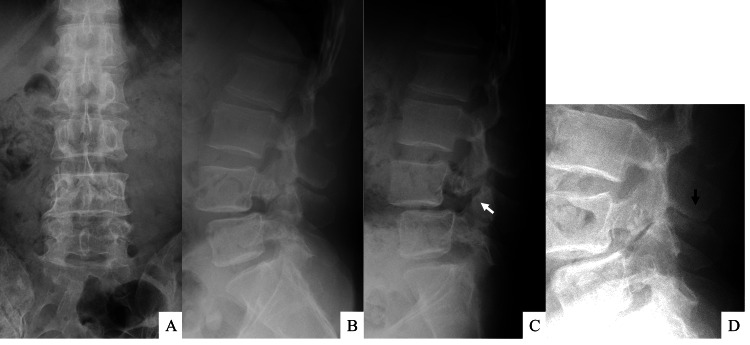
Preoperative lumbar X-rays Plain radiographs demonstrate the presence of spondylolytic spondylolisthesis at L4 on an anteroposterior (A) and lateral (B) view. Dynamic radiographs illustrate hypermobility at L4 pars on flexion (C; white arrow) and the phenomenon of "kissing" spinous processes at L3-L4 on extension (D; black arrow).

**Figure 2:**
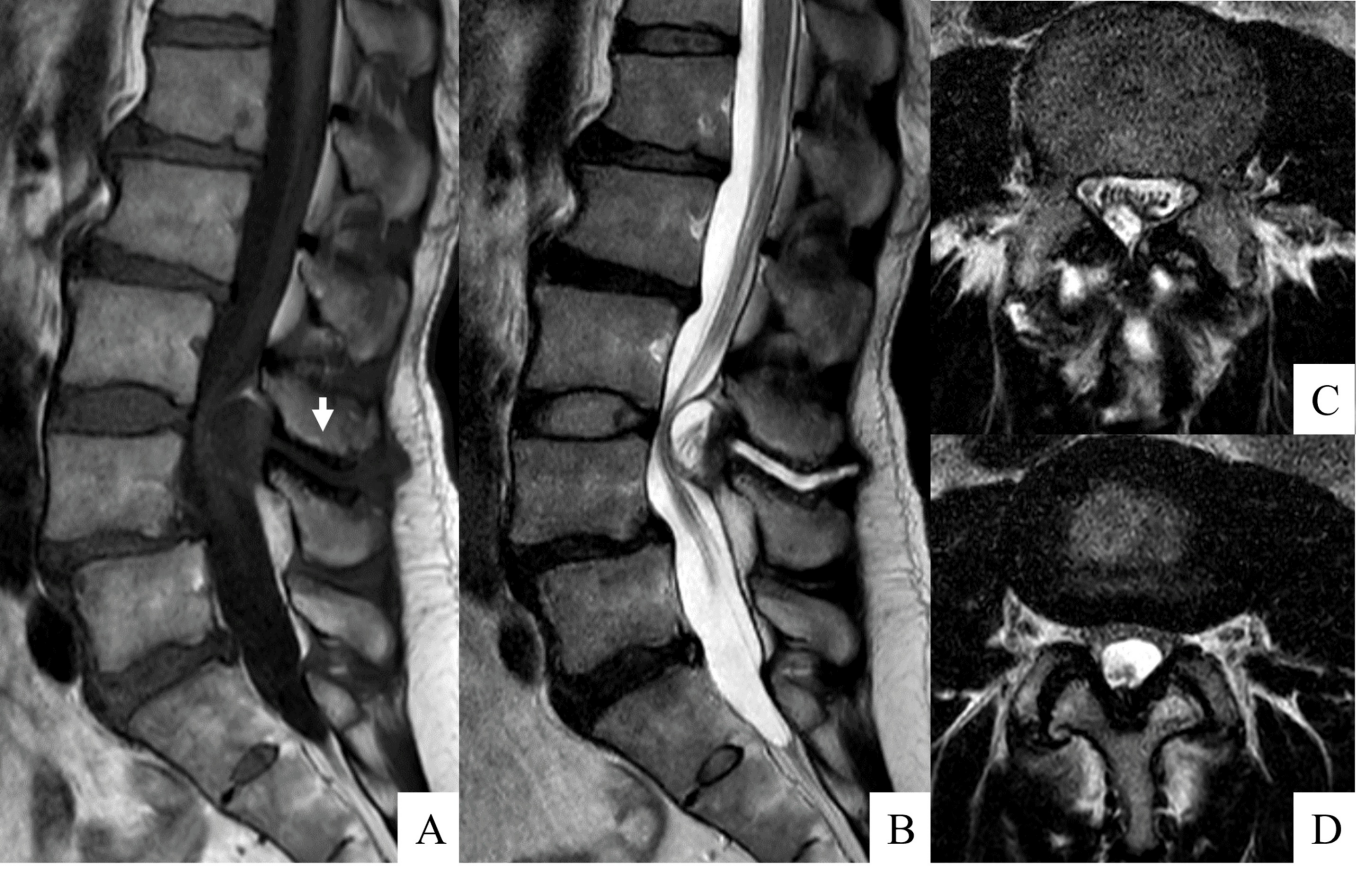
Preoperative magnetic resonance imaging Magnetic resonance imaging on T1 sagittal view shows the presence of a low intensity area (A; white arrow). Those on T2 sagittal and axial views show a high intensity area (B, C) within the L3-L4 interspinous space, accompanied by the development of an intraspinal cyst. The T2 axial view at L3-L4 disc level shows a central canal stenosis due to the cyst (D).

**Figure 3:**
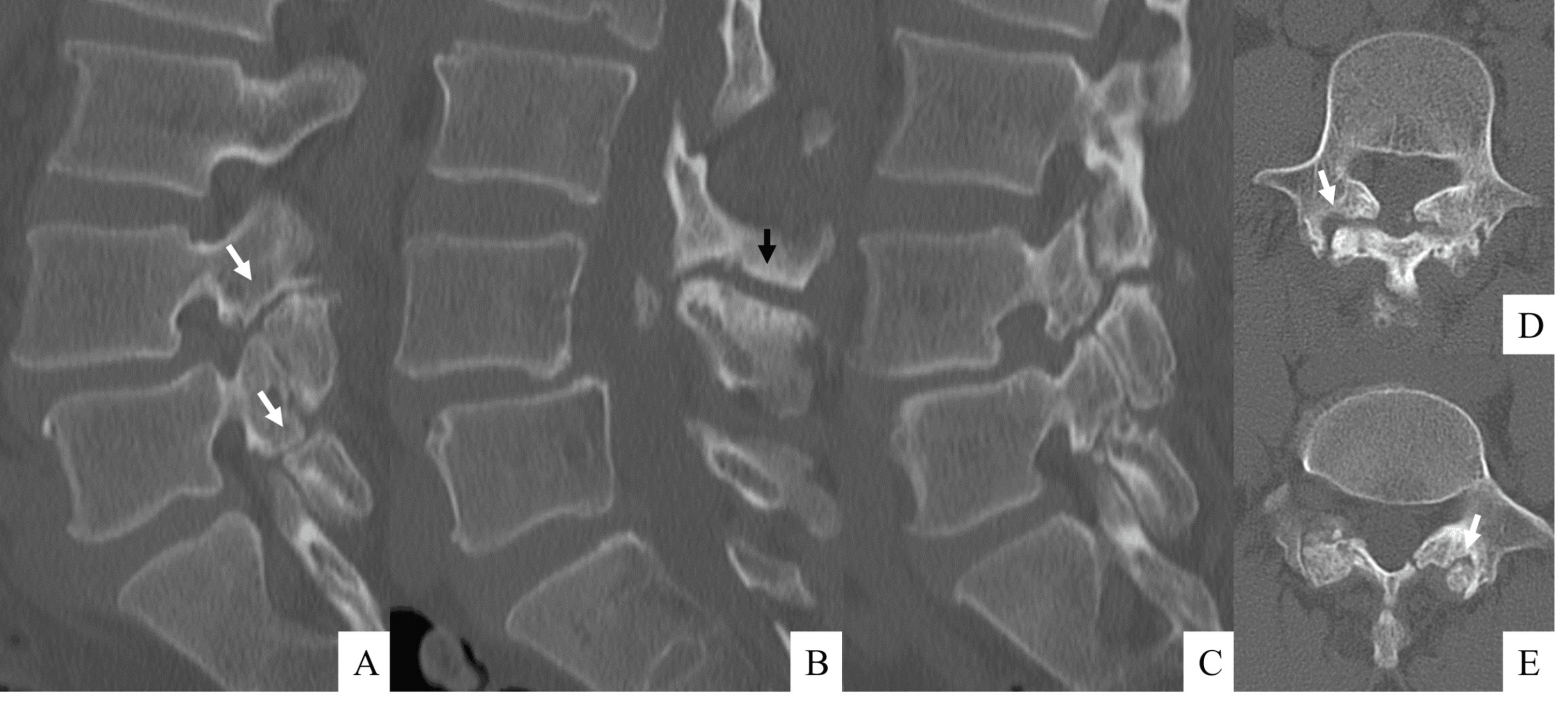
Preoperative computed tomography images A computed tomography scan reveals the presence of spondylolysis both at L4 with spondylolisthesis and at L5 with minimal slippage (A-C; from the right parasagittal to the left parasagittal view, D, E; L4 and L5 axial view, respectively. The white arrows indicate spondylosis.). Additionally, the scan demonstrated evidence of bony sclerosis and erosion between the L3 and L4 spinous processes (B; black arrow).

Radiographic examination revealed Meyerding grade 1 spondylolytic spondylolisthesis at L4 on lateral radiographs (Figure 1). Dynamic radiographs showed hypermobility at L4 pars with increased L4 slippage on flexion and "kissing" spinous processes at L3-L4 on extension. MRI showed T1-low and T2-high signal changes in the L3-L4 interspinous space along with an intraspinal cyst (Figure 2). CT revealed spondylolysis both at L4 with spondylolisthesis and at L5 with minimal slippage. Additionally, the scan showed evidence of bony sclerosis and erosion between the L3 and L4 spinous processes (Figure 3). These findings led to the diagnosis of Baastrup's disease with associated extradural cyst, L4 spondylolisthesis, and L5 spondylolysis. Written informed consent was obtained from the patient, and the institutional review board of Osaka City Juso Hospital approved this study (approved number: 2024-A6).

Surgery

**Figure 4:**
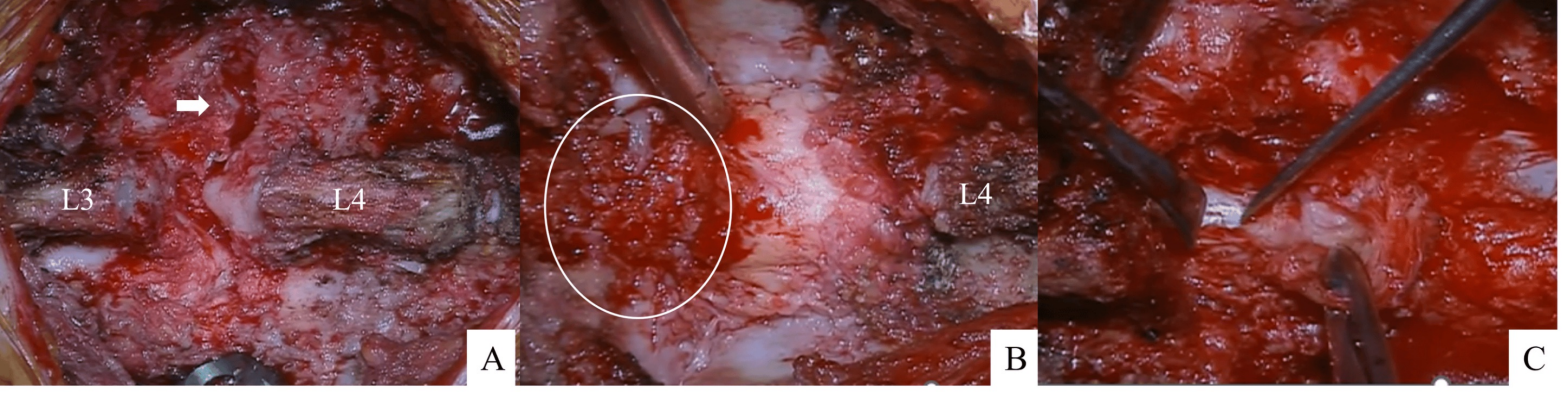
Intraoperative pictures (A) Figure after dissection of paraspinal muscles and insertion of pedicle screws; the interspinous ligament of L3-L4 had already been torn, and the serous fluid that had accumulated in the same area was aspirated. The interspinous area, the epidural cyst, and the L4 spondylolysis (white arrow) are in communication with each other. (B) Figure after the resection of the L4 lamina; the cyst in the L3-L4 intervertebral space, following serous aspiration, was observed ventral to the ligamentum flavum (white circle). (C) The cyst was excised along with the ligamentum flavum.

**Figure 5:**
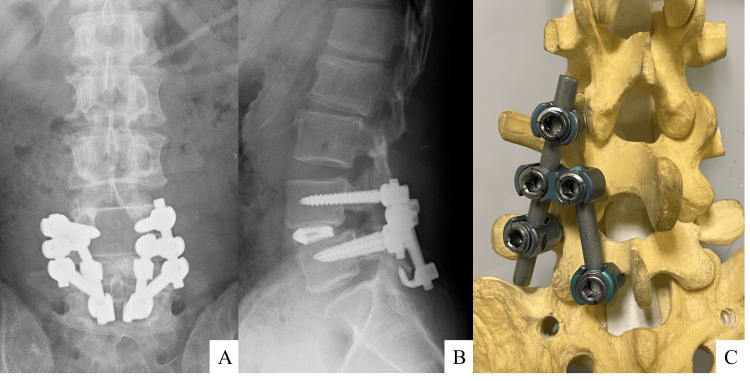
Postoperative lumbar X-rays and surgical schema Plain radiographs demonstrate the connection of the constructs to the posterior interbody fusion at L4-L5 with the direct repair of the L5 pars using lamina hooks on both the anteroposterior (A) and lateral (B) views. The components are interconnected using angled open connectors and additional rods (C).

We performed L4-L5 PLIF with cyst resection and direct repair of the pars at L5. The L3-L4 interspinous ligament was already torn, leaving only a remnant, with a serous effusion in the same area (Figure 4). The lumbar spondylolysis at L4 was contiguous with a cyst lasting from the interspinous to the epidural space. After the paraspinal muscles were dissected, the L4 lamina was unstable enough to move with the beating of the dura mater. Following this, the decision was taken to remove the L4 lamina, which resulted in the removal of the ligamentum flavum along with the cyst. The next step was PLIF with the cages after insertion of pedicle screws and rods between L4 and L5 (Stryker, Kalamazoo, MI). Finally, the L5 pars defect was repaired with the lamina hook system following decortication around the pars. Onlay grafting of morselized bone was performed. An angled open connector and another rod were used to lightly connect the PLIF construct to the lamina hook; compression was applied between the L4 and L5 pedicle screws and between the connectors and hooks, respectively, to complete the process (Figure 5).

Histopathology

**Figure 6:**
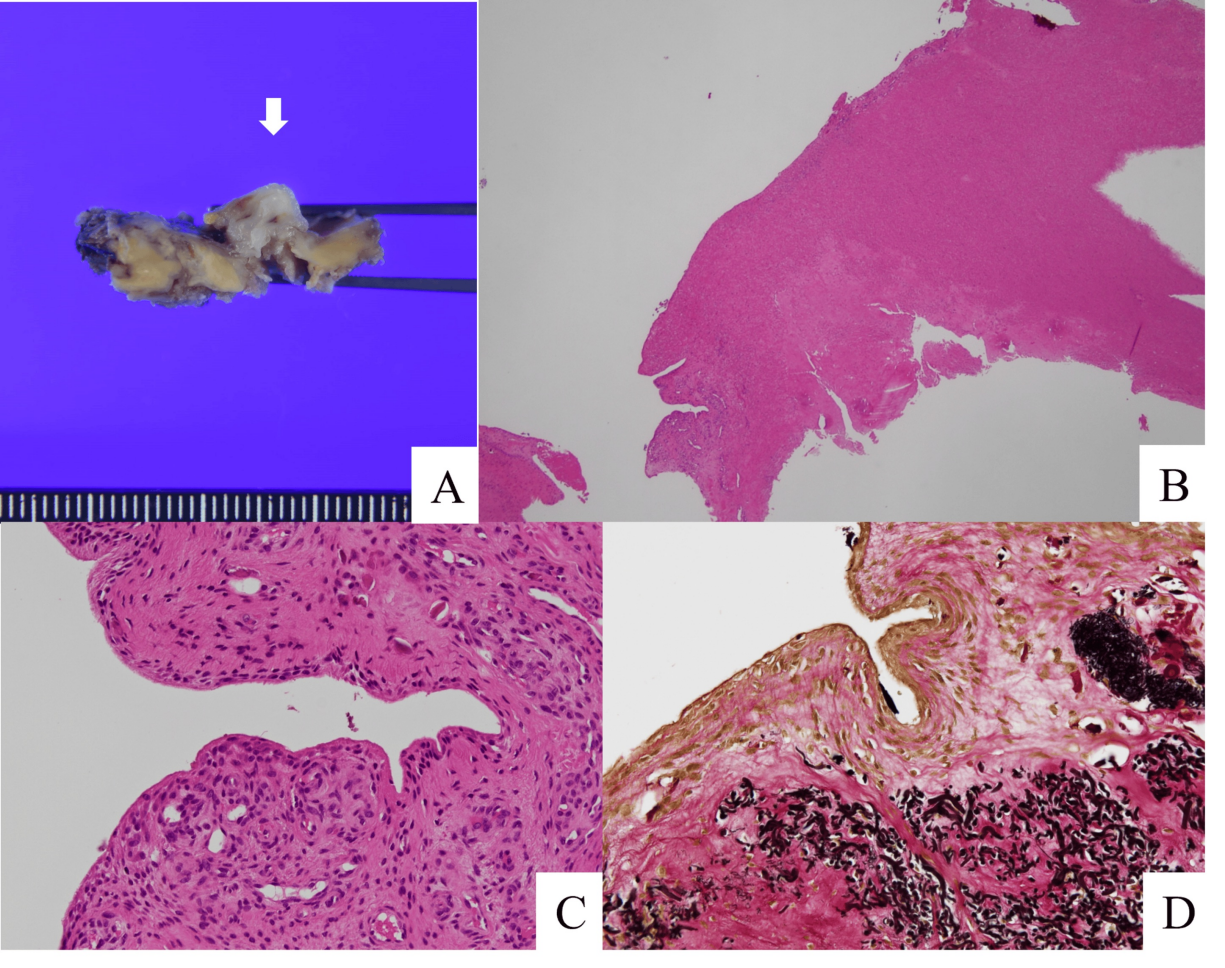
Histopathological findings of the cyst Macroscopic photographs illustrate the cyst wall (white arrow) adhering to the ventral side of the ligamentum flavum (A). Microphotographs, stained with hematoxylin and eosin (B; low power field, C; high power field) and Elastica van Gieson (D), demonstrate the cyst wall covered by synovial surface cells.

Macroscopic photographs showed the cyst wall adhering to the ventral side of the ligamentum flavum. However, the border between the ligamentum flavum and the cyst wall was clear. Microphotographs showed the cyst wall covered by synovial surface cells; a finding consistent with a synovial cyst (Figure 6).

Post-operative course

**Figure 7:**
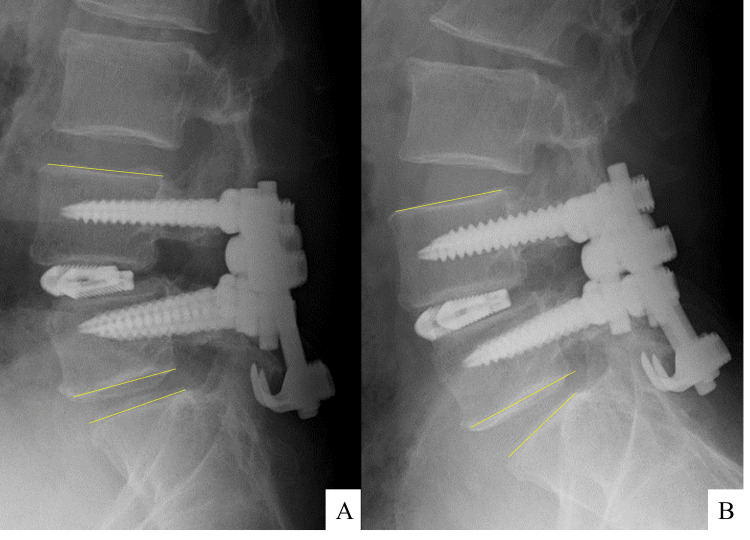
Dynamic X-rays one year after surgery Dynamic radiographs show no angular motion in flexion (A) and extension (B) at L4-L5, with motion preserved at L5-S1 and interbody bony fusion achieved exclusively at L4-L5.

The patient's neurological symptoms and intermittent claudication improved immediately after surgery, and he was discharged on the 12th day. No recurrence of lower back symptoms occurred, and the VAS score for low back and leg pain was 0 both at one and two years. The JOA score for low back pain was 28/29 at two years. Dynamic X-ray revealed that there was bony fusion on the interbody at L4-L5, while maintaining mobility at L5-S1 (Figure 7). In addition, CT revealed that bony fusion has achieved the posterior part of the pars of L5.

## Discussion

It has been observed that cystic lesions tend to form in hypermobile joints [9]. Regarding the spine, it has been put forward that facet joint cyst development may be associated with intervertebral instability [10]. The normal spine is mainly mobile at the facet joints. The presence of intervertebral instability, in conjunction with inflammation and synovial proliferation, gives rise to the formation of facet cysts at these joints. This is typically unilateral and manifests on the lateral of the dural canal. On the other hand, patients with Baastrup's disease may have greater interspinous mobility, leading to bursal cyst formation and synovial hyperplasia between the spinous processes. This could extend extradurally, compressing the dural canal from the dorsal side and causing central canal stenosis.

Based on past reports of Baastrup's disease with lumbar spondylolysis or spondylolisthesis, the formation of cysts is frequently present at the upper spinal level of the spondylolysis, as observed in the present case [4,11-13]. Additionally, there have been multiple reports of cases exhibiting similarities to the present case, wherein spondylolysis, interspinous process, and cyst are in communication with one another [4,12]. Lumbar spondylolysis can result in hypermobility at the affected level. If the site of interspinous ligament damage at the upper level can be communicated with, the vicious cycle of interspinous ligament damage and spinal hypermobility may lead to the development of Baastrup’s disease. In the presented case, the epidural cyst formation might result from hypermobility due to interspinous ligament dysfunction and spondylolysis, in addition to aging and occupation.

In the meanwhile, only one case of Baastrup's disease with associated multiple lumbar spondylolysis has been documented in which posterolateral lumbar fusion at two spinal levels was indicated [14]. Spinal fusion on multiple levels has been regarded as a prevalent surgical intervention for the treatment of multiple lumbar spondylolysis or spondylolisthesis [7]. Some reports indicate that conservative therapy may be an appropriate treatment option for one of them, but not the continuous one [15]. There is a paucity of literature on using PLIF and direct repair of lumbar spondylolysis combined. Given that epidural cysts spreading from Baastrup's disease occur at a single level and instability is involved, interbody fusion may be a suitable treatment for the affected level. However, we propose that direct repair of the pars may be indicated for another level of spondylolysis. In the present case, we aimed to achieve stabilization at L5 pars, in addition to L4-L5 interbody fusion. The pain’s etiology might not primarily reside in defects of the L5 pars, but rather in L4 spondylolisthesis and Baastrup’s disease. Consequently, the necessity to target solid bone fusion at L5 was not deemed essential.

Regarding technicalities in surgery, the use of an offset hook to connect the screw-rod construct in posterior fusion on the cephalad level and the screw-hook construct in direct repair of the pars on the caudal level appears to be a viable approach. However, it is not a straightforward process to connect the rod of the posterior fusion construct to the hook by simply extending it in a straight caudal direction. In particular, it is anticipated to be challenging in larger patients due to the insufficient offset length of the hook or caudal orientation of the outside offset of the hook, when fitted to the lamina at the spondylolysis level. A specific surgical plan is needed for the constructs connection between that of PLIF and the direct repair of the pars.

We have solved these connection difficulties by using an angled open connector. This has been used to connect the iliac screw to the posterior fixation rod. The advantages of the presented plan are that it allows separate compression forces to be applied to the PLIF and the repaired pars, and the repaired force can be applied to the pars orthogonal to the spondylolysis. In addition, there is no difficulty in connection due to body size.

## Conclusions

This case demonstrates the rare coexistence of Baastrup's disease, lumbar spondylolysis, and an epidural cyst resulting in cauda equina and nerve root symptoms. The surgical intervention, which comprised a PLIF at the affected level and direct pars repair at the adjacent level, was successful in alleviating symptoms while preserving lumbar mobility. The findings of this case suggest that avoiding multiple interbody fusions and using a combined fixation strategy may optimize clinical outcomes. They would reduce the patient burden and healthcare costs. The use of an angled open connector provided stable reconstruction, ensuring adequate compression at both the fusion and repair sites. Further studies are needed to validate this approach in similar cases.
